# A Preoperative Analytical Model for Patient-Specific Impingement Analysis in Total Hip Arthroplasty

**DOI:** 10.1155/2019/6293916

**Published:** 2019-07-01

**Authors:** Yolanda Gu, Jim Pierrepont, Catherine Stambouzou, Qing Li, Jonathan Baré

**Affiliations:** ^1^School of Aerospace, The University of Sydney, Mechanical and Mechatronic Engineering, Building J07, Sydney, NSW 2006, Australia; ^2^Optimized Ortho, 17 Bridge Street, Pymble, NSW 2073, Australia; ^3^Melbourne Orthopaedic Group, 33 The Avenue, Windsor, VIC 3191, Australia

## Abstract

Prosthetic impingement is important to consider during total hip arthroplasty planning to minimise the risk of joint instability. Modelling impingement preoperatively can assist in defining the required component alignment for each individual. We developed an analytical impingement model utilising a combination of mathematical calculations and an automated computational simulation to determine the risk of prosthetic impingement. The model assesses cup inclination and anteversion angles that are associated with prosthetic impingement using patient-specific inputs, such as stem anteversion, planned implant types, and target Range of Motion (ROM). The analysed results are presented as a range of cup inclination and anteversion angles over which a colour map indicates an impingement-free safe zone in green and impingement risk zones in red. A validation of the model demonstrates accuracy within +/- 1.4° of cup inclination and anteversion. The study further investigated the impact of changes in stem anteversion, femoral head size, and head offset on prosthetic impingement, as an example of the application of the model.

## 1. Introduction

Dislocation is one of the most common complications leading to revision surgery after Total Hip Arthroplasty (THA), accounting for almost one-quarter (21%) of all revision procedures [1]. Component mal-positioning has long been recognised as a major cause of dislocation [2–5]. Although spontaneous dislocation can occur due to poor soft tissue balance, prosthetic impingement, due to mal-positioned components, commonly precedes dislocation.

In a cup retrieval study performed by Marchetti et al., 80% of cups revised due to dislocation showed evidence of prosthetic impingement [6]. Recurrent prosthetic impingement between the femoral neck and acetabular liner can cause severe component wear due to the high stress concentration at the impingement site, producing wear debris [7]. This wear debris may result in an increased risk of osteolysis [8]. In Marchetti et al.'s study, prosthetic impingement was observed in all retrieval groups including loosening, infection, osteolysis and miscellaneous cases, highlighting the importance of an impingement-free motion to the clinical outcome of THA. Due to the numerous undesirable effects of early impingement on the stability and longevity of the prosthetic hip, it is deemed necessary to place the components in a position that avoids prosthetic impingement whilst allowing maximum hip range of motion (ROM).

Acetabular “safe zones” have been introduced by various authors to assist with cup alignment and to minimise post-operative complications. Well-known safe zones, such as those published by Lewinnek [4], Esposito et al. [9], and Callanan et al. [10] are often used as gold standard in THA. However, despite the implementation of these “safe zones”, postoperative dislocation rates remain the same [11]. In contemporary clinical studies, combined anteversion of the acetabular and femoral components, and not cup orientation alone, has been correlated with dislocation [12–15]. These more recent investigations have emphasised a need for an individualised target for cup orientation rather than a generic range applicable to all patients.

Motion capture, laboratorial simulators, and computational models are common methods of measuring range of motion of the hip [16–18]. These traditional methods can provide accurate analyses of the ROM of the hip with live subjects, generic bone models, or patient-specific bone models. However, these analyses are resource- and time-consuming and require significant setup. It is for these reasons that the above-mentioned methods are unviable for preoperative or intraoperative investigation on a large scale.

An analytical ROM model has been proposed by Yoshimine et.al. [19] and Hisatome & Doi [2] to calculate theoretical prosthetic impingement of the hip. Analytical models are faster to run and require less prework compared to traditional methods. However, analytical models are limited by their simplicity and key assumptions limit the accuracy of the model. The ROM model described in this study utilises a combination of computational simulations and analytical methods, which are accurate and efficient to run.

The aim of this study is to develop and validate a robust and accurate analytical model to assess the cup inclination and anteversion angles that are associated with prosthetic impingement using patient-specific inputs.

## 2. Method

### 2.1. Model Development

Hisatome and Doi proposed a mathematical formula to calculate the theoretical range of motion using seven factors including head radius (r), cup depth (d), cup inclination (*α*) and anteversion (*β*), stem neck angle from the transverse plane (a), stem anteversion (b), and neck width at the impingement level (n) [2]. The first six factors are defined from implant design drawings and measurements. The last factor, neck width at the impingement level, varies for different stems and at different impingement positions. Hisatome and Doi assume all stem necks are cylindrical in shape, i.e., that neck width is a constant value at all stem positions. However, the neck width varies significantly in modern trapezoidal stem designs, and the assumption of a constant neck width will significantly affect the model accuracy. The model presented in this study improves on Hisatome and Doi's work by considering neck width as a variable that differs for different stem types and impingement positions.

Hisatome provided a mathematical formula to calculate the prosthetic hip range of motion in certain activities, i.e., pure flexion, extension, internal rotation at 90° flexion, and external rotation. The impingement model in this study allows for customised inputs to define the target ROM test conditions. The proposed model offers two combined target ROM conditions: (1) user-defined degree of Internal Rotation (IR) at any Flexion (FL) and (2) user-defined degree of External Rotation (ER) at any Extension (EX). The IR_FL test is associated with anterior prosthetic impingement in flexion, and the ER_EX test is associated with posterior prosthetic impingement in extension. The derivation of how to calculate the cup orientations that satisfy the user-defined target ROM (IR_FL and ER_EX) is detailed in the Appendix.

**Figure 1:**
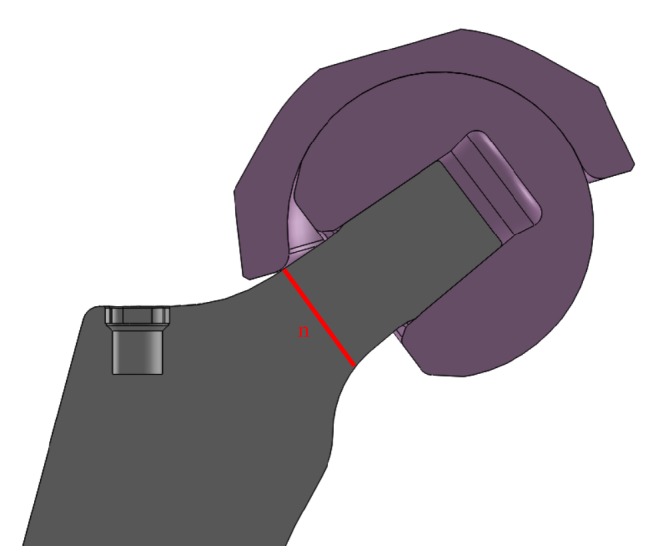
Stem neck width (n) at the impingement level.

An automated computational simulation is used to calculate the stem neck width (n) at the impingement level (Figure 1) in Solidworks (Dassault Systèmes, US). The 3D geometry of the acetabular and stem components are imported into Solidworks. The centre of rotation of the acetabular and stem components are placed at the origin of the assembly. The stem component is placed at −40° to 60° of anteversion in 20° increments and 6° of natural femur adduction. The stem component is simulated to perform flexion/extension and then internal rotation/external rotation. The acetabular component can rotate about the stem component in a range of 0° to 60° inclination and anteversion. The cup orientation is measured using Murray's radiographic definitions [20]. The automated simulation records the cup inclination and anteversion angles, and the stem neck width when any collision is detected. The results are saved in a database for use in the proposed model.

**Figure 2:**
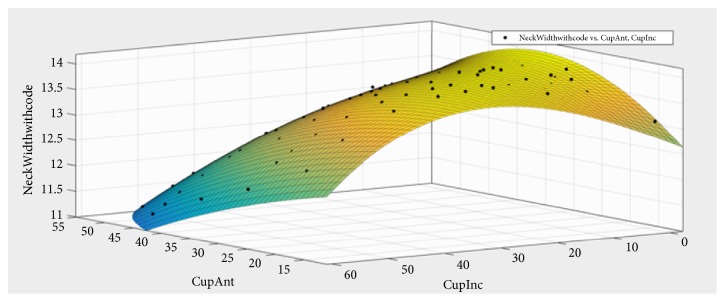
Surface interpolation of stem neck width.

Even with an automated simulation, and running only discrete values of stem anteversion, this is a time-consuming process. In order to run the impingement analysis at any stem anteversion, ‘Thin Plate Spline Interpolation' was used to calculate the neck width at all cup and stem positions, utilising the simulated neck width values. The simulated data was plotted with cup inclination and anteversion on the X and Y axes, and neck width on the Z axis in Matlab (Mathworks, US) (Figure 2). Through the interpolated surface, the stem neck width can be calculated for any given cup and stem position in all cases with the same implant type. By utilising the neck width simulator and surface interpolation combined, the neck width at any given cup and stem position can be simulated robustly and accurately. The simulated neck width can then be placed in the formula given in the Appendix and cup orientations that satisfy the target ROM conditions can be calculated. The cup inclination and anteversion which fulfil the target ROM conditions are plotted as impingement boundaries. For ease of visualisation, the area that satisfies the ROM conditions is displayed in green and the area that does not satisfy the ROM conditions is displayed in red.

**Figure 3:**
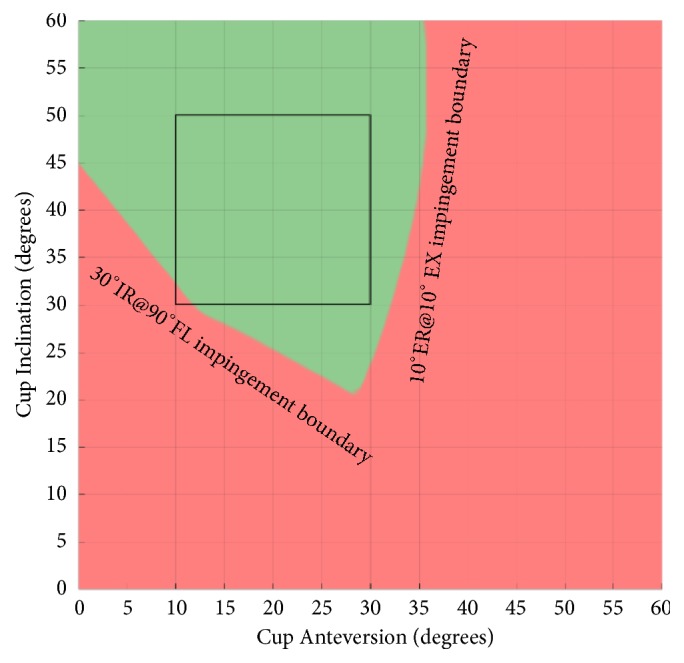
Impingement plot at 30° IR @ 90° FL and 10° ER @ 10° EX.

The proposed impingement model generates two impingement boundaries for any given set of target ROM conditions (IR@FL and ER@EX).  Figure 3 illustrates an example of the output of the model with impingement testing condition of 30°IR@90°FL and 10°ER@10°EX. The diagonal curve represents the 30°IR@90°FL impingement boundary. Cup orientations on the left-hand side of this boundary are at risk of prosthetic impingement in flexion. The vertical curve represents the 10°ER@10°EX impingement boundary. Cup orientations on the right-hand side of this boundary are at risk of prosthetic impingement in extension. The green cup orientations between these two boundaries satisfy the impingement conditions tested, and no impingement is detected. The cup orientations in red do not satisfy the impingement testing conditions as impingement is detected. A square black box is drawn on the graph for ease of comparison to the coloured area. The box highlights an area with 10° to 30° of cup anteversion and 30° to 50° of cup inclination which overlaps with commonly accepted cup positions [4, 10, 21].

Using the formula proposed in the Appendix, the cup orientations that satisfy any target ROM conditions can be calculated. The proposed model was investigated with the target ROM of 30°IR@90°FL and 10°ER@10°EX as an example. In order to test the application of the model in preoperative planning scenarios, three parameters (stem anteversion, femoral head size, and femoral head offset) were investigated to see how each parameter affects the prosthetic ROM. All other input parameters were set to the default values: neck-shaft angle = 125°, stem flexion = 0°, and stem adduction = 6°.

### 2.2. Model Validation

An alternative CAD model was used to verify that the proposed analytical model can accurately calculate the cup inclination and anteversion at impingement. The worst-case implant combinations that would provide the least accurate results were selected to validate the proposed model.

**Figure 4:**
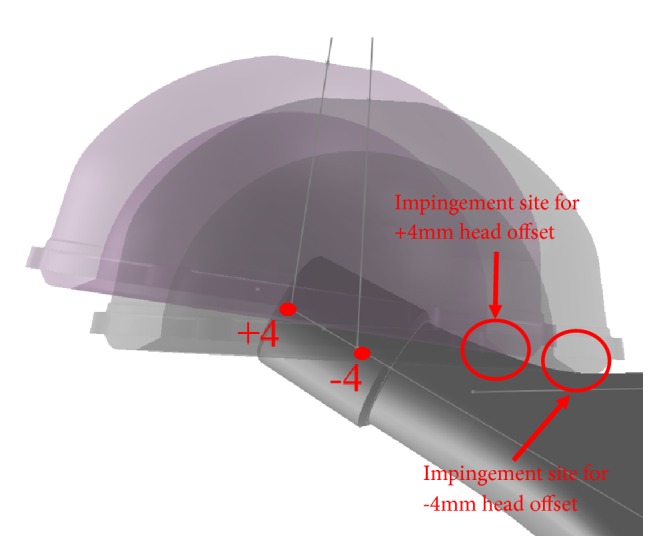
40mm polyethylene liner engaging at -4mm head offset compares to engaging at +4mm offset.

The worst-case implant size was determined with a combination of maximum head size which engaged with the shortest head offset. This resulted in the impingement to occur at the most lateral position of the stem (i.e., closer to the stem shoulder and therefore with the greatest difference in stem neck geometry (Figure 4)). This gives results furthest from the simulated impingement boundaries and therefore is the worst case for the accuracy test. The proposed model utilises the impingement boundaries in the neck width database, which was generated using 20° increments of stem anteversion, and then interpolated the impingement boundaries for other stem anteversions using the Thin Plate Spline Interpolation. Stem anteversions which would result in maximum deviation on the interpolated solution were selected for testing. It was hypothesised that the maximum deviation on the interpolated solution lies at the point which is the maximum distance away from any two exact solutions from the interpolation. The stem anteversion was placed at −40°, −20°, 0°, 20°, 40°, and 60° in the neck width database, and thus stem anteversions −30°, −10°, 10°, 30°, and 50° were selected for the accuracy test.

The implant geometries were imported into Solidworks and placed at the positions defined above. The liner was free to rotate about its centre of rotation. Using the interference detection function in Solidworks, the cup inclination and anteversion at which the acetabular component impinges with the stem can be recorded. The recorded cup inclination and anteversion in Solidworks were compared with the simulated results to determine the accuracy of the proposed model.

## 3. Results

The accuracy of the analytical model was assessed against the CAD model with worst case implant combinations. The cup orientation boundaries that were created by the proposed model and the CAD model were compared. The results show the maximum difference between the two models is 1.4° in both cup inclination and anteversion (Table 1).

In order to show how the neck width can vary at different impingement levels on the neck, four different stems were analysed within the neck width database. As shown in Table 2, the stem neck width varies significantly with different impingement locations. This suggests that using a constant neck width value is too simplistic to provide an accurate impingement analysis. The proposed model utilised a combination of an automated simulator and surface interpolation to create a database containing the neck width of the stem. Substituting the neck width database into the mathematical formula allowed the quick analysis of the prosthetic impingement model. By gaining computational efficiency in this way, the model can be used as an accurate preoperative planning tool or as a general tool to study how implant parameters impact the prosthetic ROM.

**Figure 5:**
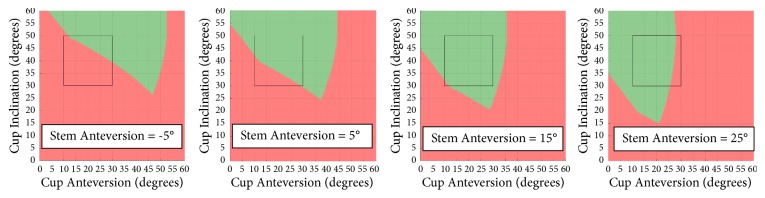
Impingement with different stem anteversion for Stem 2.

**Figure 6:**
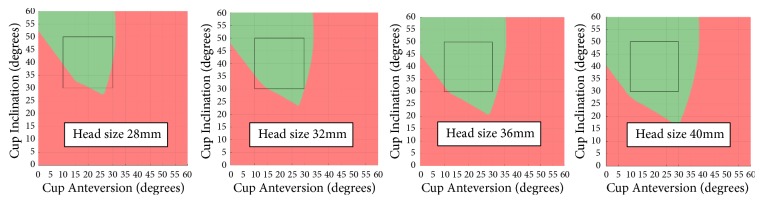
Impingement with different head sizes. At stem anteversion of 10° for stem 2.

**Figure 7:**
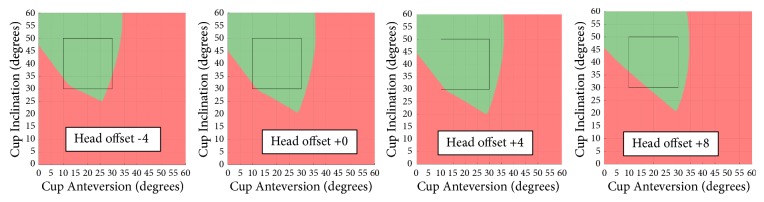
Impingement with different head offset for stem 2.

Figures 5–7 demonstrate how the prosthetic impingement can be impacted by stem anteversion, femoral head size, and femoral head offset using Stem 2 from Table 2 as an example. Similar trends can be observed with other types of stems which have a trapezoidal neck design.

As can been seen from Figure 5, as the stem anteversion increases, the impingement-free (green) zone shifts towards the low cup anteversion area, suggesting high stem anteversion must be combined with low cup anteversion in order to reduce the risk of prosthetic impingement, and vice versa. There is no significant difference in the area of the green zone at each stem anteversion. This finding agrees with the combined anteversion concept which states radiographic cup anteversion and stem anteversion are linearly correlate [2, 22, 23]. This further highlights the point that an acetabular safe zone should not be considered independent of stem anteversion.

The impingement safe zone generated by the proposed model is also affected by femoral head size. As the femoral head increases, the green zone gets larger, suggesting that a larger femoral head results in more cup component positions which satisfy the impingement testing condition (Figure 6), when all other parameters are kept constant. This agrees with previous studies that suggest that larger femoral head sizes allow for a wider range of acceptable implant orientations [24]. With a 40 mm head, the impingement-free area increases significantly compared to smaller heads. Other factors, such as wear rate and trunnionosis, should also be taken into account when selecting head sizes. Based on the implants assessed in this study, a 28 mm head should be avoided where possible as the impingement-free area decreases significantly.

**Figure 8:**
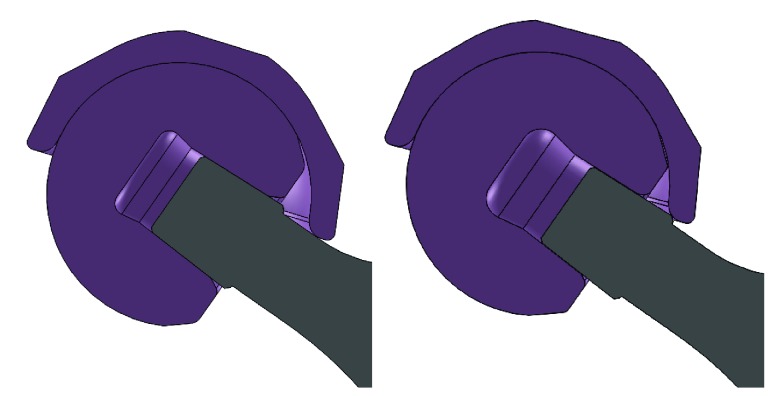
(Left) Liner impinges at stem neck with a +4mm offset head. (Right) Liner impinges at stem trunnion with a +8mm offset head.

Femoral head offset is a common parameter to consider when planning patient's leg length, offset, or soft tissue tension during THA. However, it can be overlooked during impingement analysis. The proposed model allows the impact of different head offsets on the prosthetic impingement to be considered. In the case of the stem tested (Stem 2), short (-4mm) and extra-long (+8mm) head offsets showed a smaller impingement-free zone (green area) compared to neutral (+0mm) and long (+4mm) head offsets (Figure 7), especially in the common cup orientation range (highlighted in black box). The femoral head offset is related to the stem neck width at the impingement level. Generally speaking, the shorter the offset, the wider the neck width at the impingement site, and consequently the smaller the hip range of motion (earlier impingement). However, in the case of extra-long (+8) head offset, impingement occurs at the level of the stem trunnion, which is usually wider than the stem neck. This results in extra-long head offset being less favourable than standard or long head options when trying to maximise prosthetic ROM (Figure 8).

## 4. Discussion

The present study introduced an analytical model for analysing prosthetic impingement in THA. The model involves a mathematical formula in combination with an automated neck width simulator which allows the model to take into account implant-specific neck width variations to accurately determine optimal cup orientations that are free from risk of prosthetic impingement. The model requires a one-off pre-generation of the neck width profile database for the stem of interest using the automated neck width simulator prior to the use of the model. Combined with the pre-generated neck width database and the proposed formula, the model is able to provide a zone of cup orientations that satisfy any user defined flexion and extension testing conditions, combined with any internal rotation or external rotation. The output of the model is presented as a colour map for ease of visualisation.

Even though the proposed model is capable of testing the prosthetic impingement at any user defined target ROM conditions, the question of which target ROM suits each individual patient is yet to be solved. Different target ROMs have been suggested by different groups. Incavo et al. studied passive ROM of eight cadaveric hips and suggested subjects can reach an average of 20° in extension and 24° in external rotation [25]. Similar cadaveric studies conducted by Miki et al. reported passive movement of the femur can reach up to 113° in flexion, 75° of internal rotation, 34° in extension and 36° of external rotation [26]. Only single movement and no combined movement of the femoral component were conducted in both studies. Yoshimine et al. [19] provided their combined motion ROM formula with 45° of internal rotation at 90° flexion, and Hisatome & Doi's [2] model is built based on 60° of internal rotation at 90° flexion. Both models use 30° of extension for their posterior impingement test; no combined extension and external rotation was tested. The combined motion tested in this study (30°IR@90°FL and 10°ER@10°EX) is smaller than suggested by previous authors for the following two reasons: (1) the neck width simulated from the actual stem geometry in our model is much larger than 10 mm (used in both Hisatome and Yoshimine's model). This results in fewer cup orientations that can satisfy the target ROM in our model. This also highlights the sensitivity of the stem neck width on the available ROM. The neck width of the actual stem should be used for more accurate results. (2) The previous studies suggested target ROMs were based on the results from cadaveric hips or intraoperative navigation data which did not isolate the femoral movement; i.e., pelvic movement was not taken into account during measurement of the femoral ROM. This results in overestimation of the femur movement as some of the movement observed should be attributed to the pelvis. The proposed model can accommodate different combined target ROM and was tested with 30° IR @ 90° FL, 10° ER @ 10° EX. Having customisable target ROM conditions enables the tool to accommodate inputs and test conditions on a patient-specific basis.

Similar to findings from previous studies, our model also highlights the importance of considering combined anteversion of the acetabular and femoral components. Widmer provided a simplified formula to achieve optimal ROM which suggests the sum of cup anteversion and 0.7 times the stem anteversion should be equal to 37° [22]. Similarly, Hisatome and Doi used their model and suggested cup anteversion and 0.7 times the stem anteversion should equal to 42° [2]. Other authors such as Jolles et al. and Dorr et al. suggested target ranges for combined anteversion which are 40°-60° and 25°-50°, respectively [12, 23]. Despite the various mathematical formulae or guidelines recommended in these studies, there is still a lack of general consensus of an optimal combined version. This is because the optimal combined version is multifactorial. As suggested in this study, stem version, head size, neck offset, and stem types (specifically neck width and neck angle) can all impact which cup orientations satisfy the targeted ROM. The impingement-free cup orientations determined by this model are customised for each individual and for the implant types/sizes modelled. The proposed analytical model allows different implants and positions to be input into the model and robustly calculates the range of impingement-free cup orientations.

There are a few limitations to the proposed model. First, the neck width simulator requires the specific implant geometry to be analysed prior to use. This limits the model to be used only with the implants available in the pregenerated database. More implants can be added into the database upon availability of the implant geometry. Secondly, the combined motion provided in this model is limited to the two mentioned above (IR@FL and ER@EX). Abduction and adduction of the femur were not considered in this model as it was believed that the amount of abduction and adduction was small during daily activities such as sitting and walking, in comparison to the other movements. Lastly, it is acknowledged that the functional outcome of a THA is multi-factorial and not only based on risk of prosthetic impingement. The cause of other phenomena, such as bony impingement, contact joint force, and component wear, will also significantly impact the functional outcome and longevity of the THA [9, 15, 27]. The proposed model should not be used in isolation, but rather, it should be used in combination with other analysis tools for preoperative THA planning.

This study describes an analytical model that determines a cup orientation “safe zone” for avoiding prosthetic impingement, based on accurately derived implant parameters. The model tests combined rotations of flexion/internal rotation and extension/external rotation, the limits of which can be customised. The model has demonstrated improved accuracy over other published impingement models and can be used as an investigational tool to assess the impact of varying implant parameters, in additon to preoperative planning in THA.

## Figures and Tables

**Figure 9:**
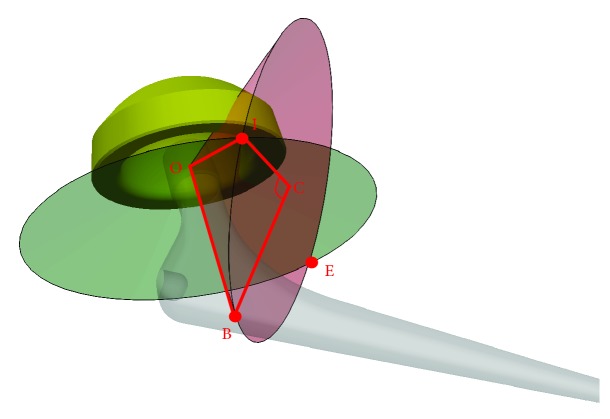
Cup cone and stem cone overlap. *∠*BCI is the maximum internal rotation angle at the defined flexion angle.

**Figure 10:**
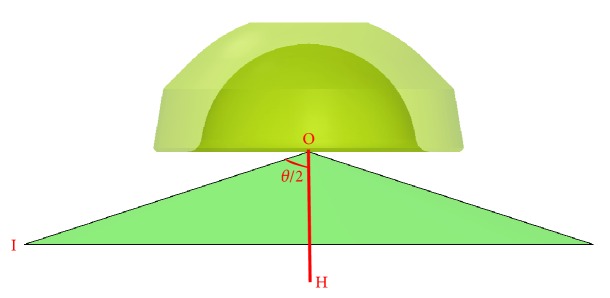
Cup cone.

**Figure 11:**
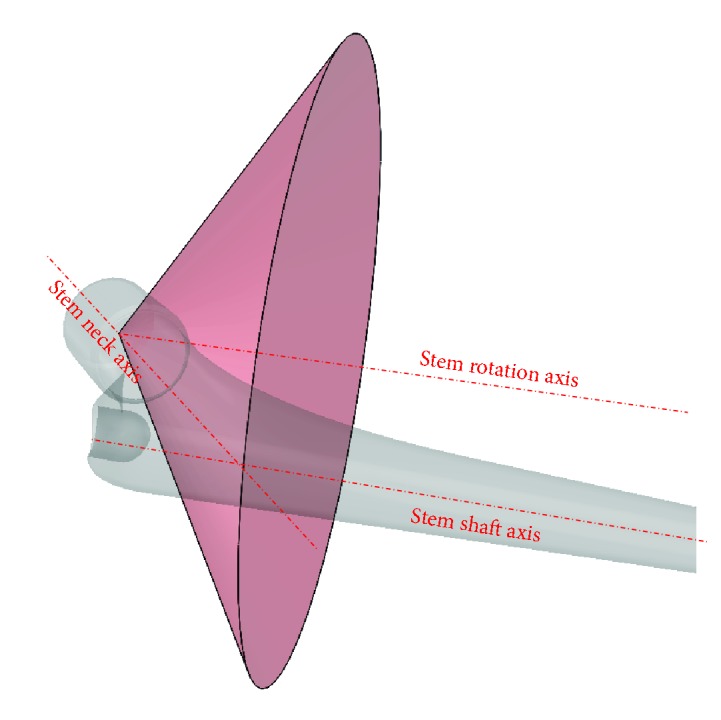
Stem cone.

**Table 1:** Maximum difference in cup orientation, at impingement in flexion and impingement in extension, between the proposed impingement model and an independent CAD model.

	Cup Anteversion	Cup Inclination
Maximum difference in flexion	1.1°	1.4°
Maximum difference in extension	1.4°	1.0°

**Table 2:** Neck width range for four stems investigated in this study.

Stems	Neck width range in mm (Avg)
Stem 1	11.3 – 22.2 (14.7)
Stem 2	10.9 – 17.3 (13.5)
Stem 3	11.0 – 18.2 (13.7)
Stem 4	10.9 – 20.3 (13.7)

## Data Availability

The data used to support the findings is contained within the article.

## Appendix

The concept of creating the IR_FL formula is to calculate the maximum rotation of the stem at a defined degree of flexion via finding the intersection point I (INTRflx, INTRfly, INTRflz) of the cup rotation cone and stem rotation cone (Figure 9). The cup rotation cone is a right circular cone with a unit generatrix which is formed by rotating the stem component inside the cup. The orientation of the cup cone changes with changes in cup inclination and anteversion angle (Figure 10). By applying vector analysis to the cup cone, we can get the following two equations:

(A.1) I=INTRflx2+INTRfly2+INTRflz2=1

(A.2) INTRflx∗sin⁡β+INTRfly∗sin⁡α∗cos⁡β−INTRflz∗cos⁡α∗cos⁡β=cos⁡θ2

where *α* and *β* are cup inclination and anteversion, respectively. *θ* is the oscillation angle which can be calculated by *θ* = 360° - 2*∗*sin^−1^(n/2r)-(180°- 2*∗*sin^−1^((r-d)*∗*r)), where n is the neck width, r is the head radius, and d is the head centre offset from the liner centre.

The stem rotation cone is another right circular cone with a unit generatrix formed by rotating the stem at defined flexion angle (Figure 11). By applying vector analysis to the stem cone, we can get the following equation:

(A.3) $$\begin{array}{c} INTRflx*\sin\left(\;FL\;\right)-INTRflz*\cos\left(\;FL\;\right)=\sin\left(\;a\;\right) \end{array}$$

where a is the angle between stem neck axis to transverse plane.

The maximum internal rotation angle is equal to *∠BCI*, where B is the initial position of the stem and C is the base centre of the stem cone (Figure 9). By solving (A.1), (A.2), and (A.3) simultaneously, the coordinates of I (INTRflx, INTRfly, INTRflz) can be solved. Subsequently, *∠BCI* can be calculated and this is the maximum internal rotation (Max_IR) of the stem before impingement occurs.

(A.4) Max_IR=∠BCI=acos⁡INTRflx−sin⁡a∗sin⁡FL∗sin⁡a∗sin⁡FL−cos⁡a∗sin⁡b∗cos⁡FL−sin⁡a∗sin⁡FL+INTRfly∗cos⁡a∗cos⁡b+INTRflz+sin⁡a∗cos⁡FL∗−cos⁡FL∗sin⁡a−sin⁡FL∗cos⁡a∗sin⁡b+sin⁡a∗cos⁡FL×cos⁡a ∧2−1

where a is the angle between stem neck axis to transverse plane and b is the angle between stem neck axis to coronal plane which projected onto transverse plane (stem anteversion).

The same concept can be applied to calculate the maximum external rotation of the stem at any defined extension angle.

(A.5) Max_ER=acos⁡EXTRexx+sin⁡a∗sin⁡EX∗−sin⁡a∗sin⁡EX−cos⁡EX∗sin⁡b∗cos⁡a+sin⁡a∗sin⁡EX+EXTRexy∗cos⁡a∗cos⁡b+EXTRexz+sin⁡a∗cos⁡EX∗−cos⁡EX∗sin⁡a+sin⁡EX∗sin⁡b∗cos⁡a+sin⁡a∗cos⁡EX×cos⁡a ∧2−1

where Max_ER is the target maximum external rotation defined by the user and EX is the target extension angle defined by the user. EXTRexx, EXTRexy, and EXTRexz are the coordinates of intersection point of the cup rotation cone and stem rotation cone when stem is in extension, a is the angle between stem neck axis to transverse plane, and b is the angle between stem neck axis to coronal plane which projected onto transverse plane (stem anteversion).
